# A Finger Vein Liveness Detection System Based on Multi-Scale Spatial-Temporal Map and Light-ViT Model

**DOI:** 10.3390/s23249637

**Published:** 2023-12-05

**Authors:** Liukui Chen, Tengwen Guo, Li Li, Haiyang Jiang, Wenfu Luo, Zuojin Li

**Affiliations:** 1 College of Intelligent Technology and Engineering, Chongqing University of Science & Technology, Chongqing 401331, China; newque_chen@cqust.edu.cn (L.C.); 2022208013@cqust.edu.cn (T.G.); 2020208031@cqust.edu.cn (H.J.); 2022208010@cqust.edu.cn (W.L.); 2 Wuhan Maritime Communication Research Institute, Wuhan 430202, China; csiclily2023@126.com

**Keywords:** finger vein liveness detection, MSTmap, Light-ViT

## Abstract

Prosthetic attack is a problem that must be prevented in current finger vein recognition applications. To solve this problem, a finger vein liveness detection system was established in this study. The system begins by capturing short-term static finger vein videos using uniform near-infrared lighting. Subsequently, it employs Gabor filters without a direct-current (DC) component for vein area segmentation. The vein area is then divided into blocks to compute a multi-scale spatial–temporal map (MSTmap), which facilitates the extraction of coarse liveness features. Finally, these features are trained for refinement and used to predict liveness detection results with the proposed Light Vision Transformer (Light-ViT) model, which is equipped with an enhanced Light-ViT backbone, meticulously designed by interleaving multiple MN blocks and Light-ViT blocks, ensuring improved performance in the task. This architecture effectively balances the learning of local image features, controls network parameter complexity, and substantially improves the accuracy of liveness detection. The accuracy of the Light-ViT model was verified to be 99.63% on a self-made living/prosthetic finger vein video dataset. This proposed system can also be directly applied to the finger vein recognition terminal after the model is made lightweight.

## 1. Introduction

Biometric-based identification technology [1,2] has seen widespread adoption; however, information security is of paramount importance, with a particularly notable challenge being posed by prosthetic attacks. Instances of prosthetic attacks targeting human faces and fingerprints have been well-documented [3]. Despite the inherent vitality of finger vein recognition, the risk of prosthetic attacks persists when original vein line information is compromised. Although there have been no reported cases of prosthetic attacks resulting from the exposure of original vein pattern information, Tome et al. [4] have conducted simulations of prosthetic vein attacks capable of deceiving existing hand vein recognition systems with a notably high success rate. The detection of liveness in a biometric recognition system has emerged as a pivotal aspect for its secure deployment, and the assessment of liveness in finger vein recognition has consequently become a fundamental prerequisite and a focal point of research [5].

Compared to other body surface features, finger veins are in vivo characteristics that do not leave marks during daily activities. While theft of original vein pattern information is challenging, there remains a risk of leakage of this information. Firstly, an existing company’s hand vein recognition technology has demonstrated the collection and extraction of hand vein pattern information under visible light conditions [6]. Secondly, there are risks associated with the unauthorized collection of hand vein patterns using self-made equipment. To address these risks, further research on in vivo detection technology is necessary for the application of this technology [7]. In this paper, we propose using a short-length video of a finger vein to power a living vein recognition system. Compared with one static vein image, the extracted vein video provides more spatial–temporal information and can provide more robust live features for detection.

The key to liveness detection technology lies in the extraction of liveness features. Traditional image processing methods and deep learning models have been used in vein liveness detection. In particular, neural networks have better performance than artificially designed features in static image texture feature and dynamic video spatial–temporal feature extraction [8,9], and deep learning methods have become a hot research field in biometric liveness detection. Inspired by remote photoplethysmography (rPPG) [10] and multi-scale spatial–temporal graphs [11], combined with the Vision Transformer (ViT) architecture [12], this paper proposes an improved Light-ViT network for finger vein short-frame video liveness detection.

The remaining sections of this paper are organized as follows. Section 2 provides an overview of the relevant research work on finger vein liveness detection. Section 3 introduces the various modules of the finger vein liveness detection system, such as finger vein video acquisition, vein segmentation and block selection, MSTmap generation, and the proposed Light-ViT model design. Section 4 presents the prosthetic creation method and examines the proposed model’s performance through comparative experiments. Finally, conclusions are given in Section 5.

## 2. Related Research

In recent years, prosthesis attack has gradually become the main means to break the biometric identification system. This type of attack involves using printed pictures, prostheses, videos, and other means to deceive a biometric identification system and authenticate an unauthorized person as a real user. Attackers use tools such as pictures, prostheses, and videos as presentation attack tools [13]. Liveness detection technology for general biometrics utilizes individuals’ physiological characteristics. For example, liveness fingerprint detection can be based on finger temperature, sweating, electrical conductivity, and other information. Liveness face detection can be based on head movement, breathing, red-eye effects, and other information. Liveness iris detection relies on iris vibration characteristics, motion information from eyelashes and eyelids, and the dynamic response of the pupil to changes in visible light intensity. The corresponding liveness detection procedure can be divided into traditional liveness feature extraction and deep learning liveness feature extraction methods for single static images and multi-frame videos. The primary attack method for finger vein recognition technology is print attack. The attacker can use a stolen finger vein image, which they print onto materials such as paper or film or attach to a real finger to complete the biometric forgery attack.

In 2011, Maatta et al. [14] proposed a texture-based analysis method, which uses color texture as a presentation attack detection (PAD) clue. By extracting complementary low-level features from different color spaces to describe the joint texture information of brightness and chrominance channels, combined with discrete Fourier analysis, the characteristics of energy distribution in the spectrum of prosthesis images are used as PAD clues, which have a high accuracy for printed prosthesis images.

In 2012, Chingovska et al. [15] proposed a PAD method for texture analysis of photo attacks based on weighted neighborhood difference quantization local binary patterns, which are used to combat attacks using printed images and video replays. It achieved good results on three facial anti-fraud databases.

In 2015, the GRIP-PRIAMUS team [16] used the fact that a printer always prints in a certain direction to find a larger energy relative to the vertical direction from the frequency-domain transformation so as to detect whether a living vein was present. In the first finger vein deception-attack prevention attempt, a method based on the use of local descriptors was proposed. Firstly, local phase quantization (LPQ) was used to encode the spatial domain information for the complete image, and then the local binary pattern (LBP) was used to extract the texture features of the segmented vein region image. After fusing the features of the two, they were sent to the support vector machine (SVM) for classification.

In 2018, Fang et al. [17] proposed a novel finger vein recognition method. This method initially utilizes total variation regularization techniques to decompose the raw finger vein image into two components: structure and noise. Subsequently, block local binary pattern descriptors are employed to encode the structural and noise information in the decomposed image components. Finally, a cascaded support vector machine model is used for classification to achieve precise finger vein recognition. This approach not only reduces the impact of image noise and other interfering factors on recognition but also enhances recognition accuracy and robustness.

In 2019, Lee et al. [18] utilized the rPPG principle to effectively extract liveness features from finger veins by using Fourier analysis and wavelet transform. These features were then classified using SVM to achieve vein liveness detection. This technique extracts frequency data by enhancing the vein video in multiple directions and widths. The resulting frequency signal is obtained by means of wavelet transformation and enables the detection of biometric attacks in a non-contact, accurate, and robust manner.

While this frequency-domain-based approach has advantages in terms of ease of implementation, it has several drawbacks, including high computational complexity, sensitivity to noise, and poor robustness. Moreover, it relies on pre-defined feature parameters, limiting its generalization capabilities. Deep learning methods have been successfully applied to across various domains, especially convolutional neural networks (CNNs), which have witnessed significant advancements in the field of computer vision, notably in biometric recognition [19]. Deep learning and its inherent feature learning capabilities have paved a new path for the development of biometric PAD systems.

In 2017, Assim et al. [20] proposed a method called GA-CNN, which combines genetic algorithms and convolutional neural networks (CNNs), resulting in significant improvements in both accuracy and sensitivity. The core idea behind this method is to optimize the hyperparameters of the neural network using genetic algorithms, thereby enhancing the model’s performance and robustness. GA-CNN performs outstandingly not just in detecting liveness veins but also in its proficiency to cope with vein images at different scales, rendering it more adaptable and practical for applications in the real world.

In 2020, Baweja et al. [21] introduced a novel approach for anomaly detection training. The absence of negative samples renders end-to-end learning impractical in one-class classification. Therefore, the authors proposed a “pseudo-negative” sample feature space, which aids the model in comprehending the decision boundaries between genuine and counterfeit samples more effectively. The pseudo-negative class is modeled using a Gaussian distribution. In contrast to other existing one-class classification (OCC) models, both the classifier and feature representation undergo end-to-end learning in this approach.

In 2020, Zeng et al. [22] devised an end-to-end model that combines fully convolutional neural networks (FCNNs) with conditional random fields (CRFs) to extract vein pattern networks for finger vein verification. In 2021, Tao et al. [23] employed mask region-based convolutional neural networks (Mask-RCNNs) to obtain precise region of interest (ROI) images and developed a softmax-based decision algorithm for verification. Gionfrida et al. [24] proposed a model for recognizing gesture sequences from video using appearance and spatial–temporal parameters of consecutive frames and combined a convolutional network with a long- and short-term memory unit, which was experimentally shown to outperform previous temporal segmentation models. Sandouka et al. [25] proposed a solution based on a transformers and generative adversarial networks (GANs) for liveness fingerprint detection, and the experimental results demonstrated that this architecture increased the adaptive mean classification accuracy from 68.52% to 83.12%. Adam et al. [26] analyzed methods for detecting fingerprint spoofing and proposed an ensemble classifier model that achieved a 2.5% reduction in error rate compared to existing methods, resulting in an accuracy rate of 90.34% for correct classification. In 2022, Zhou et al. [27] proposed a virtual sample generation method called SIFT flow-based virtual sample generation (SVSG). This method uses the scale-invariant feature transform flow (SIFT-flow) algorithm to obtain displacement matrices for each category with multiple registered samples. The process then involves extracting key displacements from each matrix, removing noisy and redundant displacements, and ultimately producing a conclusive global variation matrix. The experimental results demonstrated the efficacy of this method in enhancing the performance of single-sample finger vein recognition.

Deep-learning-based approaches leverage neural networks to learn data distributions without the need for manually designed feature extraction algorithms. Networks trained on extensive data exhibit high accuracy. However, this method has drawbacks, such as the difficulty in obtaining training data and the need to validate network generalization for specific problems. Additionally, some networks can be structurally complex, have a large number of parameters, lack real-time capabilities, and pose challenges in terms of portability to mobile platforms.

## 3. Our Method and System

**Figure 1:**
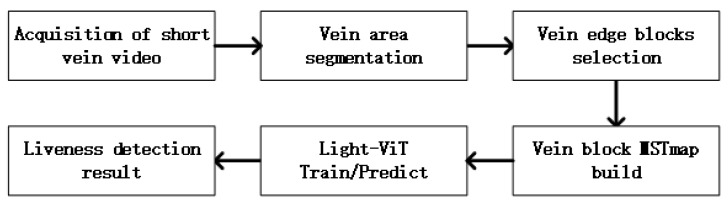
Flowchart of finger vein static short-term video liveness detection system.

In this paper, the system uses the finger vein static short-term video as the data source for in vivo detection. There is blood flow within the superficial subcutaneous vein, and there will be slight expansion and contraction, leading to slight changes in the absorption rate. During angiographic imaging, when near-infrared (NIR) light penetrates a vein, there is a slight grayscale change in the vein area, which can be detected using video image processing methods [28,29], and when there is no fluid flow through the ‘blood vessel’ in the prosthesis, there will be no grayscale change in the vein area. However, the slight grayscale transformation will be submerged in the speckle noise of near-infrared angiography. In order to solve this problem, this paper proposes an artificial shallow feature extraction with a deep learning model for finger liveness detection. The flow chart of this system is shown in Figure 1. The first step is to obtain a short-duration static finger vein video. The next step is vein area segmentation, which belongs to the preprocessing. The third step is to select and cut out small blocks on the edge of the vein. The fourth step is to construct the MSTmap for these sorted small blocks. The fifth step is training the proposed Light-ViT model with the MSTmap. The last step is to output the result of liveness detection.

**Figure 2:**
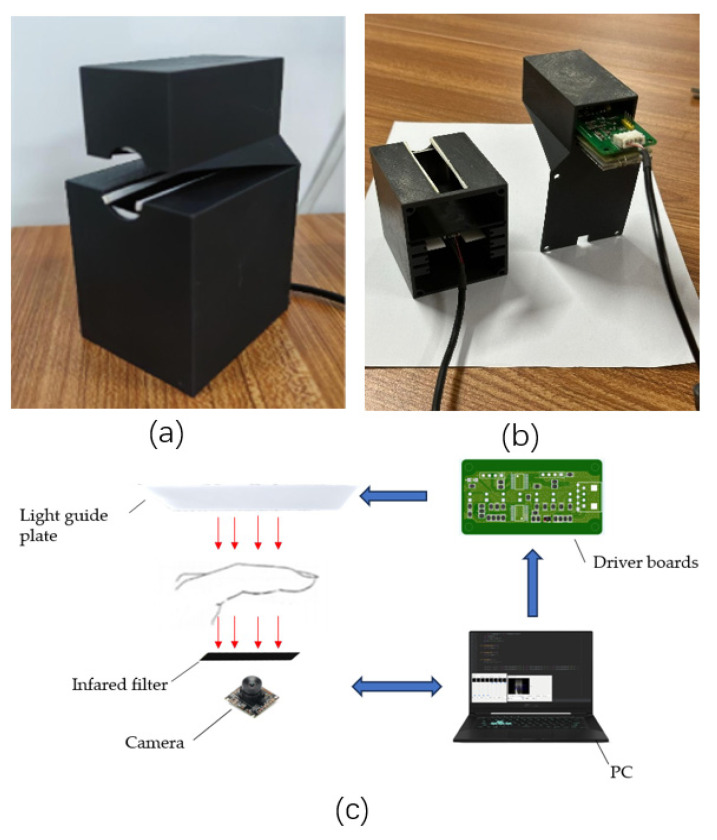
Hardware equipment: (**a**) housings image; (**b**) internal structure diagram; (**c**) acquisition process image.

Our self-made finger vein short-term video acquisition platform is shown in Figure 2. The housing is manufactured using 3D printing technology and the upper light source bracket is designed to hold the light source group securely in place. This arrangement ensures stability and uniformity of the light source. In contrast, the main body of the case is made of black opaque material and the driver circuit board adjusts the light intensity to minimize the effects of ambient light and improve the accuracy of image acquisition. An opening in the top of the housing serves as a viewing window where a finger can be placed for image acquisition. This location facilitates the extraction of the region of interest (ROI) during post-processing. A groove designed on the inside of the bottom stabilizes the position of the camera and the NIR filter.

When a user places their finger between the light source of the device and the viewing window, the image grabber starts capturing the image of the finger vein. In this process, the controller constantly monitors and analyzes the quality of the captured image, and it quickly adjusts the light intensity of the light source according to the changes in the brightness, contrast, and other parameters of the image, so that the brightness and clarity of the captured image are always kept within the preset range. The application of intelligent control can be quickly adjusted according to the actual situation to ensure that the qualities of the captured images are similar, thus improving the recognition accuracy and stability of the equipment.

### 3.1. Obtaining Short-Term Static Finger Vein Images

**Figure 3:**
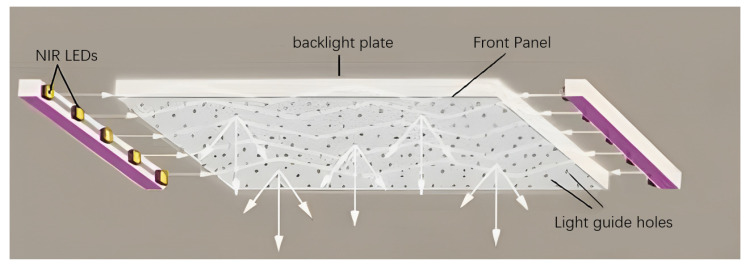
Low chart of finger vein static short-term video live detection system.

In this paper, a uniform illumination method is used to obtain a short-term static finger vein image. A light guide plate is used as the light source plate. The back and front panels of the light guide plate have a reflective layer. The near-infrared light emitted by the side near-infrared LED beads is reflected between the back plate and the front panel. After being reflected, it is emitted through the pre-distributed light guide hole of the front panel. The optical simulation software (lighttools v8.6) can be used to calculate the aperture and distribution of the light guide hole and finally form a uniform illumination, as shown in Figure 3. The advantage of using the light guide plate is to avoid overexposure caused by glare. After multiple reflections, the light passes through the small light guide hole with less energy and does not cause glare. When the traditional LED lamp bead array light source collects the vein image, if the finger does not block the lamp bead, the direct light of the lamp bead will cause a large area of overexposure when it enters the camera. The image/video of uniform near-infrared illumination transmission finger venography has the same illumination conditions. The noise distribution of each region of the finger vein image is consistent, and the later modeling is simpler and more convenient.

### 3.2. Preprocessing of Video Frames

In order to meet the real-time application of vein liveness detection and facilitate use, when collecting the finger vein video, we used the three-frame difference method [30] to extract the frames in which the fingers remained stationary in the short-duration video. The method efficiently detects moving targets and captures their subtle movement changes, eliminating the need for later multi-frame pixel-level registration of the finger vein, to reduce and prevent an excessive computational load. The user’s finger only needs to stay in the acquisition device for 1.5 s. The camera uses a high-speed camera, taking 120 frames per second, and the pixel resolution is 640 × 480. The camera collects RGB three-channel images. Although there are near-infrared (NIR) filters in front of the lens, the corresponding near-infrared contrast images can still be collected. The noise of each channel is different, which can be used to increase the signal-to-noise ratio of small grayscale changes in the vein area.

The multi-scale and multi-direction Gabor filtering method is used to segment the vein region. This paper presents a fast Gabor filter design method to remove the DC component, which is equivalent to the method in Reference [31]. The real part of the traditional Gabor filter is designed as follows:

(1) $$G\left(x,y\right)=\exp\left(-\frac{x_{1}^{2}+\gamma^{2}y_{1}^{2}}{2\sigma^{2}}\right)\left(\cos\left(2\pi \frac{x_{1}}{\lambda }+\varphi \right)-exp\left(\frac{\nu^{2}}{2}+\varphi \right)\right).$$

(2) $${\;x}_{1}=xcos\theta +ysin\theta .$$

(3) $$y_{1}=-xsin\theta +ycos\theta .$$

Here, γ  represents the aspect ratio of the space, which determines the ellipticity of the shape of the Gaussian kernel function curve. When γ=1, the shape is a positive circle; σ is the standard deviation of the Gaussian function, and its value cannot be directly set, but is related to the bandwidth. λ  is the wavelength of the sine function; φ  is a sine function phase; θ  is the rotation angle. Equations (2) and (3) illustrate that the Gabor function can be elongated in the $\left(x,y\right)\;$ plane in any direction determined by θ.

In order to quickly remove the DC component of the filter template, this paper proposes to directly calculate the mean value of the Gabor filter as the DC component removed. The formula can be expressed as follows:

(4) $$G\left(x,y\right)=\exp\left(-\frac{x_{1}^{2}+\gamma^{2}y_{1}^{2}}{2\sigma^{2}}\right)\left(\cos\left(2\pi \frac{x_{1}}{\lambda }\varphi \right)\right).$$

(5) $$G^{\prime }=G-mean\left(G\right).$$

### 3.3. Selection of Vein Edge Image Block

The segmented binary vein image is subjected to morphological corrosion operation, and the vein edge region is obtained by subtracting the corroded image from the binary original image, as shown in Equation (6):

(6) $$I_{edge}=I_{bw}-erode(I_{bw},H).$$

In Formula (6), $I_{bw}$ is the binary image segmented from the previous vein, erode is the binary image erosion operation function, and H is the morphological operator, here taking a 3 × 3 size.

### 3.4. Multi-Scale Spatial–Temporal Map Calculation

**Figure 4:**
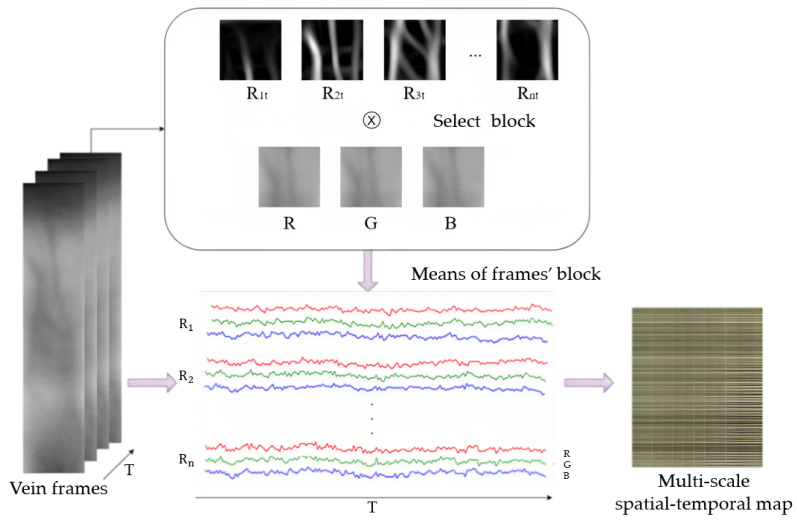
Construction of multi-scale spatial–temporal map.

In order to highlight the change in vein grayness caused by blood flow, it is more noise-resistant to observe the mean change in vein block than the gray change of one pixel. In this paper, the image blocks on the edge of the vein are selected from the root of the finger to the tip of the finger along the main vein, and p blocks are selected. The mean gray value of each frame image block in the video is taken, from which the gray change in the image block over time can be obtained. The three different color lines presented in the image represent the time-varying curve of the gray level of the extracted finger vein image in the three color channels of RGB. The gray mean value of multiple image blocks with time changes to form a time–space map, as shown in Figure 4. 

### 3.5. Build Light-ViT Model

The MSTmap of the finger vein that we constructed contains varying grayscale information while preserving features, which is challenging for prosthetics to achieve. The transformation of the static finger vein video into the MSTmap necessitates that the network possesses the capability to effectively manage long-range pixel relationships. Simultaneously, the conversion of vein edge position features requires dedicated attention to local characteristics. Convolutional neural networks (CNNs) have consistently exhibited exceptional proficiency in feature extraction [32]. However, when dealing with spatial temporal maps of multiple scales that encompass both global and local features, CNNs still exhibit limitations in comprehensive global feature extraction. In contrast, the ViT network leverages its multi-head attention mechanism to excel in local feature handling, complemented by its capacity for long-range pixel-to-pixel feature extraction through positional encoding. The ViT network has been empirically proven to deliver superior performance, yet it grapples with challenges such as large parameter scales, training complexity, and suboptimal performance on smaller datasets. Furthermore, finger vein presentation attack detection (PAD) serves as a pivotal technology in biometric identification, where precision and real-time responsiveness constitute fundamental requirements. Considering that biometric identification devices are typically compact in size and operate within the constraints of limited computational resources offered by computer chips, it becomes imperative to maintain a compact system architecture. Consequently, we introduce the Light-ViT model, which not only adeptly captures both global and local data features but also facilitates seamless integration into our system, achieving high-precision finger vein counterfeit detection at a significantly reduced cost.

The fundamental concept of the Light-ViT network involves the creation of L-ViT blocks to replace the conventional convolution used in MobileNet. The L-ViT backbone constitutes the core of Light-ViT. This network is composed of multiple MobileNet blocks (MN blocks) and L-ViT blocks stacked alternately. Specifically, the MN block employs depth-wise separable convolution operations with the aim of learning local image features while controlling the network’s parameter count, enabling better adaptation to large-scale datasets. On the other hand, the L-ViT block adopts a Transformer structure to capture global image features and integrates them with locally extracted features obtained through convolution.

**Figure 5:**
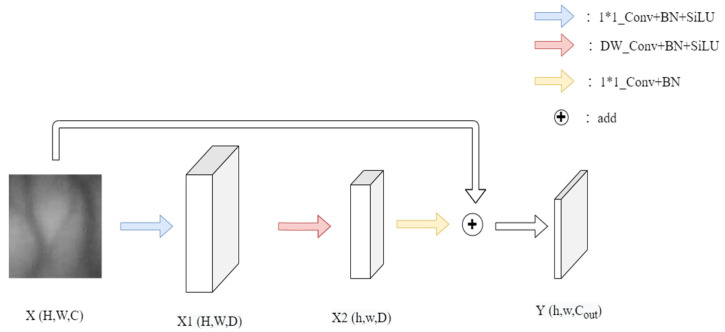
The structure of the MN block.

The MN block is a convolution module within the network used for learning image biases and local features. Its structure is illustrated in the diagram. For input features X, it initially undergoes a 1 × 1 convolution layer, effectively mapping to a higher dimension via pointwise convolution. Subsequently, it passes through a batch normalization (BN) layer and the SiLU activation function to obtain X1, where X1, and it can be adjusted based on network requirements. Following this, it undergoes group convolution, followed by another BN layer and the SiLU activation function to obtain X1. Here, T represents the stride, and adjusting this parameter governs the dimensions of the resulting tensor. After merging with the input features through a reverse residual structure, the dimensions of X1 are mapped to X2 using pointwise convolution (PW), followed by a BN layer to yield the output Y. The structure of the MN block is shown in Figure 5.

**Figure 6:**
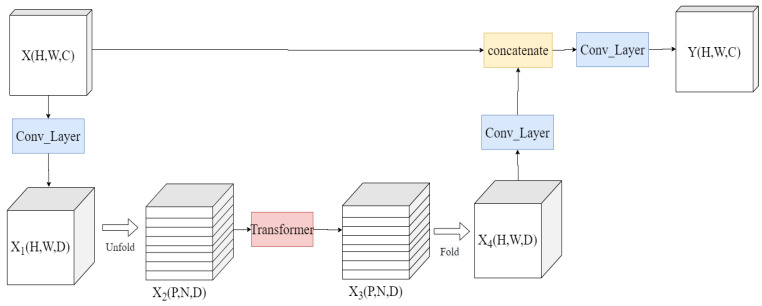
L-ViT block structure diagram.

This method has the advantage of significantly reducing the number of parameters and computational resources required for convolution, while maintaining the same convolutional kernel sensing field for the convolution kernel, as previously discussed [33]. Additionally, to equip the CNN with the capacity to learn global features, we have introduced an L-ViT module, as depicted in Figure 6. Given an input X(H,W,C), we commence by encoding the local spatial information through a 3 × 3 standard convolutional layer. Subsequently, we utilize a 1 × 1 convolutional layer to project the feature dimensions to a higher space, resulting in $X_{1}(H,W,D)$. To enable Light-ViT to acquire global representations with spatial inductive bias, we unfold $X_{1}$ into N patches and aggregate them to yield $X_{2}(P,N,D)$, where P=w∗h, with w and h denoting the width and height of a patch, and N=H∗W/P.

Subsequent spatial coding of $X_{2}$ produces $X_{3}(P,N,D)$, preserving both the ordering information among each patch and the spatial details of the pixels within each patch. Then, $X_{3\;}$ undergoes a 1 × 1 convolutional projection to return to a lower-dimensional space. It is then concatenated with X through a residual structure, followed by fusion of features using a standard convolution operation.

**Figure 7:**
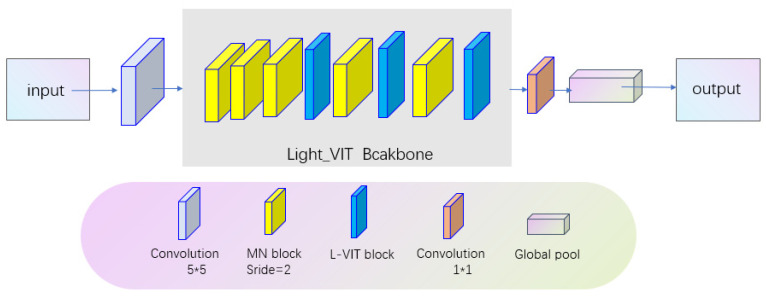
Light-ViT network structure.

The L-ViT block is primarily used to learn global features of the feature map. We employ an Unfold operation to process the input tensor, introducing positional information to the feature map while retaining the Encoder part that introduces attention mechanisms. However, we replace the Decoder part with convolution operations to adjust the size of the output feature map. The enhanced Light-ViT further strengthens the network’s understanding of images and provides more powerful performance through the comprehensive extraction and fusion of local and global features. The structure is depicted in Figure 7.

The input image first passes through a standard convolutional layer, primarily serving the purpose of generating shallow-level feature maps and adjusting the input dimensions. Subsequently, the feature maps are fed into the Light-ViT backbone, which consists of multiple MN blocks and L-ViT blocks stacked alternately. This allows the network to learn new high-level feature maps that integrate both local and global features. These features can capture image details and global information more accurately, thereby providing richer and more comprehensive information for subsequent classification tasks. Then, a 1 × 1 convolutional layer maps the feature maps into a high-dimensional space. Finally, the network’s output is obtained through global pooling and a linear layer. In global pooling, the network can perform statistical operations over the entire feature map to acquire more comprehensive and rich information. Finally, through the transformation of a linear layer, the network converts the feature map into a vector with corresponding class probability distribution.

Our proposed Light-ViT significantly reduces the demand for computational resources and greatly enhances the network’s feature learning capabilities by introducing MN blocks and improving L-ViT blocks. When integrated into the system, this lightweight network exhibits high recognition accuracy.

## 4. Experiment and Discussion

### 4.1. Introduction of Experimental Data

**Figure 8:**
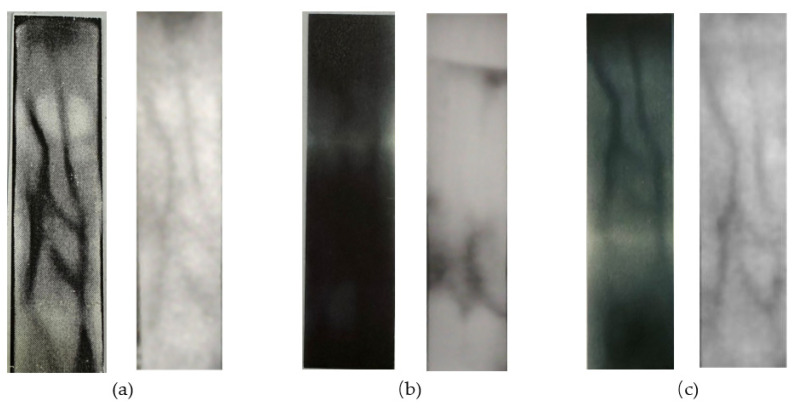
The prosthesis made of three different materials: (**a**) A4 paper; (**b**) PVC plastic; (**c**) laser film.

There is no public finger vein prosthesis or in vivo short video on the Internet at present. In this experiment, three kinds of finger vein prosthesis were made, short videos of near-infrared finger veins were collected by using self-made equipment, and simulated forgery attack experiments were carried out. The dataset consists of 400 short videos, including 200 live finger vein videos, from 100 different fingers. The prosthesis materials are divided into A4 paper, PVC plastic, and laser film. And 100 prosthesis finger vein videos were collected. These video samples cover different angles and different lighting conditions. In the experiment, the dataset was randomly divided into a training set and test set according to the ratio of 8:2, and ensures that there are no repeated samples. Therefore, the training set contains 320 videos, and the validation set contained 80 videos. The earlier methods [34] for prosthesis fabrication had suboptimal imaging quality within the data acquisition equipment introduced in this chapter. Common issues included a significant presence of noise, excessive venous contrast, and regions of excessive blurriness within the acquired images, the fabricated prosthesis, and the acquired images, as are depicted in Figure 8.

**Figure 9:**
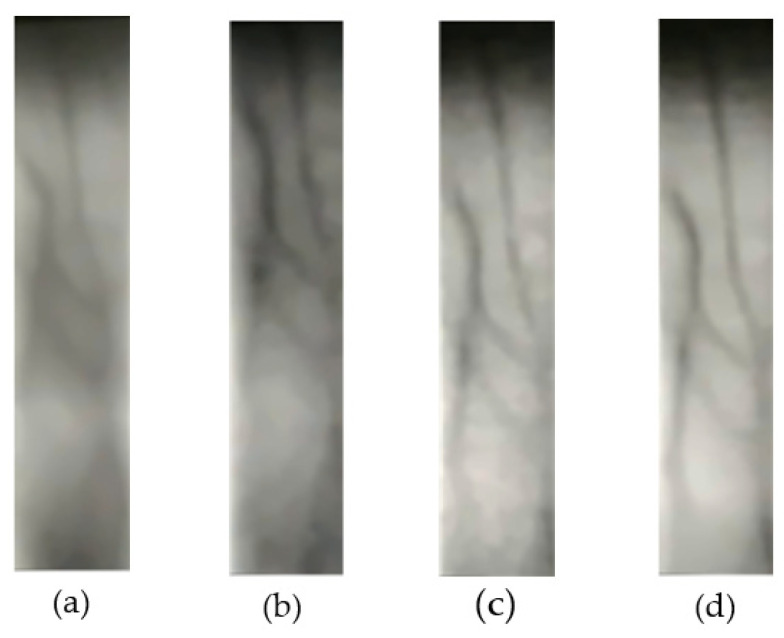
The captured images in vivo and of prosthesis: (**a**) living sample; (**b**) A4 paper; (**c**) PVC plastic; (**d**) laser printing film.

Real veins exhibit consistency in color and texture within non-venous areas. To simulate this characteristic in prostheses, we initially applied Gaussian filtering to blur real vein images and eliminate noise. Subsequently, we enhanced vein features through contrast-limited histogram equalization. Following this, we performed adaptive regional threshold segmentation, using the resulting image as a mask for applying local power-law transformations to the original image. Experimental results demonstrated the quality of the acquired images of our fabricated prostheses, as illustrated in Figure 9 below.

Before the model training, all video samples were preprocessed and converted into MSTmap representations as input data. The size of the MSTmap image was (224, 180, 3). Finally, 400 MSTmap images were sent to the network or used in the experiment.

### 4.2. Model Parameters

In the process of network training, the following parameters were set: the batch size was 32; the number of parallel tasks was 8; the number of training rounds was set to 300; and the initial learning rate was 0.0001, which was dynamically adjusted according to the cosine annealing strategy. The optimizer was the adaptive moment estimation method, and the loss function was cross entropy loss.

The loss function was used to measure the network output and label errors. The purpose of neural network back-propagation was to reduce the error between the output and labels. The main task of the network in this paper was classification. 

### 4.3. Evaluation Indicators

In order to objectively evaluate the performance of the finger vein PAD algorithm and compare the performance between the implemented algorithms, researchers usually use the relevant evaluation indicators in international standards [35]. The indicators that evaluate the detection effect of the liveness detection system usually include accuracy rate (ACR), attack presentation classification error rate (APCER), and bona fide presentation classification error rate (BPCER).

The calculation formulas of APCER and BPCER are as follows:

(7) $${APCER}_{PAIS}=1-\left(\frac{1}{N_{PAIS}}\right)\sum_{i=1}^{N_{PAS}}RES.$$

(8) $$BPCER=\frac{\sum_{i=1}^{N_{PAS}}RES}{N_{BF}}.$$

Among these, the quantity of prosthesis samples is the number of real samples. If the i sample is classified as a prosthesis, it is 1, and vice versa, it is 0. The biometric anti-counterfeiting system should pursue higher security, so the higher the APCER, the better the security of the system, taking into account the two indicators of ACR and BPCER.

### 4.4. Experiment

Initially, we transform video samples into MSTmap images to extract dynamic liveness features. Subsequently, these MSTmap images are fed into an enhanced ViT network for classification to determine if the sample represents an authentic finger vein pattern. Compared to the DFT + SVM method [13], our approach eliminates the need for intricate frequency-domain transformations and exhibits superior generalization capabilities. In contrast to the EVM + MPR method [36], our technique is not dependent on the choice of filtering frequency and showcases heightened resistance to noise interference. Moreover, when compared to the LBP + WLD method [37], our methodology more effectively harnesses the dynamic information present in videos and avoids the information loss associated with texture feature extraction. The results of the experiment are shown in Table 1.

Based on our experimental results, the liveness feature extraction method, MSTmap combined with Light-ViT classification, achieved the highest accuracy rate of 99.63%. In comparison, the DFT + SVM and EVM + MPR methods registered accuracy rates of 91.04% and 82.92%, respectively. The approach employing LBP + WLD yielded the lowest accuracy on the dataset, standing at a mere 78.75%.

The advent of lightweight networks provides crucial technical support for biometric systems. By integrating these lightweight networks into biometric systems, we can significantly reduce the consumption of computational resources while still identifying and capturing essential details within biometric features. To further assess the performance of the Light-ViT network in this task and make comparisons with other influential and representative networks in the field, this study selected the following networks for comparative analysis: (1) VGG-16 is a CNN-based image classification network comprising 16 layers of depth and the utilization of compact convolutional kernels [38]. (2) ResNet50 is a CNN-based architecture grounded in residual learning principles, capable of training networks with a depth of 50 layers while mitigating issues like gradient vanishing and degradation [39]. (3) ViT is a vision transformer-based image classification network that leverages self-attention mechanisms to capture global features, demonstrating superior computational efficiency and accuracy compared to traditional CNNs [12]. (4) MobileNetV2 is an example of a lightweight convolutional neural network that substantially reduces network parameters through techniques like depth-wise separable convolutions [40]. The accuracy of the test set is presented in Table 2.

**Figure 10:**
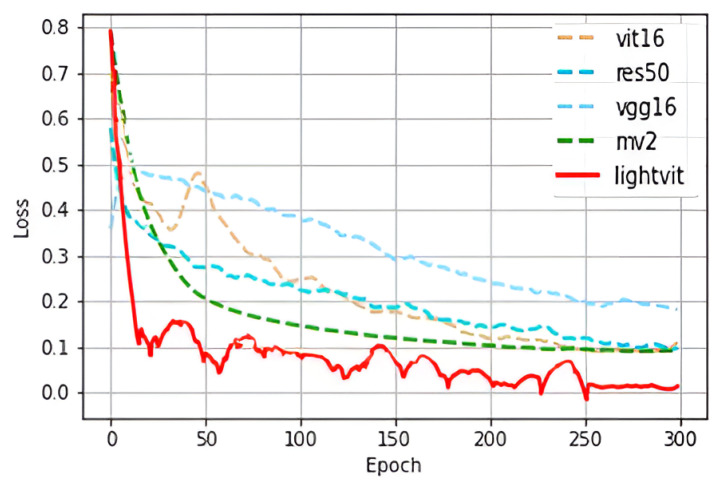
Loss curve.

From the experimental results, it can be seen that Light-ViT is optimal on the MFVD dataset with 99.63% accuracy and a 0 false acceptance rate. Among the tested networks, MobileNetV2, ResNet50, and ViT demonstrate the highest performance, achieving accuracies of 97.25%, 97.22%, and 96.87%, respectively. In contrast, VGG16 lags behind as the least effective option, achieving an accuracy of 92.47%. These results underscore the efficacy of the Light-ViT network in effectively amalgamating the strengths of both CNN and ViT architectures, enabling the learning of both global and local features within an image, consequently enhancing detection accuracy. The variation in the network loss function in the above experiment is shown in Figure 10.

The parameter and computational load for each network are detailed in Table 3. In terms of network size and computational demand, Light-ViT exhibits a notable advantage. Its lightweight architecture can significantly reduce the system’s response time.

To evaluate the influence of our network structure improvements on the final results, we replaced the L-ViT block in the Light-ViT network architecture with a standard convolution layer of size 3 × 3, modified the number of convolution channels, and removed the linear bottleneck structure, resulting in the baseline network (Basenet). The experimental results are presented in Table 4.

**Figure 11:**
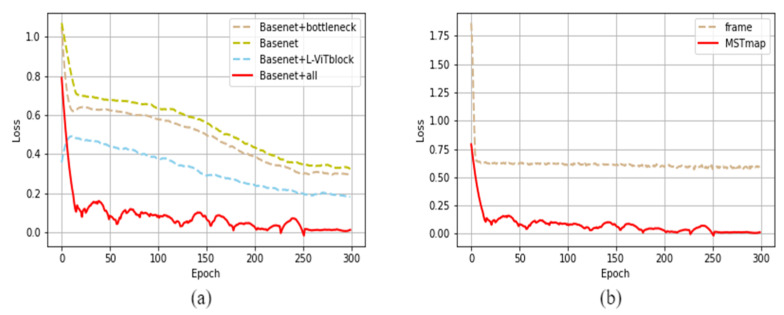
The ablation experiment loss curves of Light-ViT. (**a**) Loss curve of ablation experiment; (**b**) MSTmap experimental loss curve.

The experimental results indicate that the proposed L-ViT block significantly enhances the network’s performance. By introducing the ViT structure, the network gains the capability to learn global features. To validate the role of MSTmap in aiding subsequent network feature recognition, we designed the following comparative experiments. Firstly, we directly used frames extracted from the vein videos, randomly selecting four frames from each video sample, amounting to 7056 training images. These were then trained using the Light-ViT network. The graph below illustrates the loss curve from the ablation study. The loss plot is shown in Figure 11.

To evaluate the performance of the vein anti-counterfeiting identification system proposed in this study, which incorporates a lightweight network, we chose finger vein recognition as a specific application scenario and compared it with other representative network structures. We employed the MFVD dataset, FV-USM dataset, and VERA dataset, conducting data augmentation on them and splitting them proportionally into training and validation sets, as detailed in Table 5.

We also conducted comparative experiments on other lightweight networks, and the experiments demonstrated that our proposed Light-ViT achieved better performance on three different datasets. Table 6 is used to illustrate their comparative results with the use of ACR.

The Light-ViT network, as introduced in this paper, demonstrated commendable performance in the identification task, attaining an accuracy of 98.81% on the MFVD dataset. This achievement surpassed that of other conventional CNN and ViT networks. Notably, even though all the alternative network architectures achieved accuracy rates exceeding 95% on the MFVD dataset, the Light-ViT network distinguishes itself by offering a more compact parameter size and requiring less computational effort. These attributes render it well-suited for deployment and operation on mobile devices. Consequently, Light-ViT emerges as a neural network capable of effectively harnessing global information processing capabilities, with substantial advantages in identity recognition tasks.

## 5. Conclusions

This paper proposes a video detection system for vein pattern anti-counterfeiting recognition. In this system, we propose a lightweight network and enhance its capacity to learn global features by introducing L-ViT blocks. Additionally, we replace standard convolutions with depth-wise separable convolutions to construct MN blocks, reducing the computational cost of convolution operations and consequently reducing the network’s size and computational load. We embed this lightweight network into the system and transform video data into multi-scale spatial–temporal graphs to allow the network to extract more feature information. Experimental results demonstrate that Light-ViT offers fast computation, a compact parameter footprint, and achieves higher recognition accuracy compared to other lightweight networks. The integrated detection system with this network exhibits superior real-time performance and usability. Prosthetic assaults persist as a prominent concern in the realm of bioinformatics security. The challenge confronting finger vein vivisection lies in the emergence of more sophisticated types of attacks. Despite the outstanding capabilities demonstrated by our proposed system in experiments, we plan to conduct further research in two key areas. Firstly, we aim to enhance the device to address prosthetic attacks in various scenarios. Secondly, we will continue to strengthen the deep learning network’s ability to extract features from the MSTmap of finger veins. Our future work will also delve deeper into the field of bioinformatics security.

## Figures and Tables

**Table 1:** Comparative test of common PAD methods.

Experimental Method	ACR	APCER	BPCER
LBP + WLD	0.7875	0.325	0.2861
EVM + MPR	0.8292	0.2083	0.1583
DFT + SVM	0.9104	0.0833	0.0917
MSTmap + Light-ViT	0.9963	0	0.0037

**Table 2:** Comparative experimental results.

Network Name	ACR	APCER	BPCER
VGG16	0.9247	0.0803	0.0712
ResNet50	0.9687	0.0364	0.0367
ViT	0.9722	0.0294	0.0299
MobileNetV2	0.9725	0.0281	0.0307
Light-ViT	0.9963	0	0.0037

**Table 3:** Network parameters.

Network Name	Total Params (M)	Params Size (MB)	GFLOPS (M)
VGG-16	134.269	512.19	30,932
ResNet	23.512	89.69	8263
ViT	85.648	326.72	33,726
MobileNetV2	2.226	8.49	652.419
Light-ViT	1.107	4.22	690.217

**Table 4:** Results of ablation experiment.

Network Structure	ACR	APCER	BPCER
Basenet	0.9090	0.0928	0.1005
Basenet + L-ViT block	0.9809	0.0147	0.0239
Basenet + bottleneck	0.9287	0.0603	0.0716
Basenet + all	0.9963	0	0.0037

**Table 5:** Dataset composition.

Dataset	Amount (Picture)	Class	Proportions
MFVD	17,280	288	8:2
FV-USM	23,616	492	8:2
VERA	14,080	220	8:2

**Table 6:** Identity identification experiment results and network parameters.

Network Structure	ACR		
	MFVD	VERA	FV-USM
VGG16	0.9588	0.9172	0.9285
ResNet50	0.9687	0.9426	0.9428
ViT-16	0.9699	0.9359	0.9471
MobileNetV2	0.9684	0.9428	0.9452
Light-ViT	0.9881	0.9612	0.9702

## Data Availability

Data are contained within the article.
